# S4. ASYMMETRIC DRUG-INDUCED PARKINSONISM IS RELATED TO PSYCHOPATHOLOGY

**DOI:** 10.1093/schbul/sby018.791

**Published:** 2018-04-01

**Authors:** Lydia Pieters, P Roberto Bakker, Peter N Van Harten

**Affiliations:** 1 Psychiatric Center GGz Centraal; 2 Maastricht University, Psychiatric Centre GGz Centraal

## Abstract

**Background:**

Drug-Induced Parkinsonism (DIP) is the most common movement disorder induced by antipsychotics. The prevalence of DIP in chronic psychiatric populations ranges between 17 and 72% (1–3). Although, DIP is mostly symmetric, asymmetric DIP is reported in 18 to 54% of the patients. (4). There are no studies to the clinical relevance of asymmetric DIP. We investigated the prevalence of motor asymmetry in DIP and its relationship to the severity of psychopathology in a prospective study.

**Methods:**

In a cohort study of 207 long-stay psychiatric inpatients the prevalence of DIP was assessed at least two times (mean follow-up 1.1 year) in each patient (5). DIP was assessed with the Unified Parkinson Disease Rating Scale (UPDRS) and the prevalence of persistent DIP was 56.2%. Patients with at least one time parkinsonism in the upper/lower limb(s) were included for analyses. Asymmetry of parkinsonism was calculated with the symmetry index (Figure 1). A cut-off value of ≥ 0,20 was used for the definition of asymmetric DIP. Multilevel mixed models were built to explore the relationship between asymmetry in DIP and the severity of psychopathology, measured on the Clinical Global Impression-Schizophrenia scale severity index (CGI-SCH SI).

**Results:**

In a cohort study of 207 long-stay psychiatric inpatients the prevalence of DIP was assessed at least two times (mean follow-up 1.1 year) in each patient (5). DIP was assessed with the Unified Parkinson Disease Rating Scale (UPDRS) and the prevalence of persistent DIP was 56.2%. Patients with at least one time parkinsonism in the upper/lower limb(s) were included for analyses. Asymmetry of parkinsonism was calculated with the symmetry index (Figure 1). A cut-off value of ≥ 0,20 was used for the definition of asymmetric DIP. Multilevel mixed models were built to explore the relationship between asymmetry in DIP and the severity of psychopathology, measured on the Clinical Global Impression-Schizophrenia scale severity index (CGI-SCH SI).

**Discussion:**

DIP is asymmetric in 1 of 5 patients. Therefore, the clinical rule that Parkinson’s disease always starts asymmetrically and such may be helpful to differentiate between Parkinson’s disease and DIP is not valid. Asymmetric presentation of DIP is of clinical relevance as it is related to the severity of psychopathology. Asymmetric DIP may alert the clinician of more severe psychopathology. Replication is indicated to examine the robustness of the relationship.

**References:**

1. Modestin J, Wehrli MV, Stephan PL, Agarwalla P. Evolution of neuroleptic-induced extrapyramidal syndromes under long-term neuroleptic treatment. Schizophr Res. 2008; 100(1–3): 97–107.

2. Janno S, Holi M, Tuisku K, Wahlbeck K. Prevalence of Neuroleptic-Induced Movement Disorders in Chronic Schizophrenia Inpatients. Am J Psychiatry. 2004; 161(1): 160–163.

3. Harten PN Van, Matroos GE, Hoek HW. The prevalence of tardive dystonia, tardive dyskinesia, parkinsonism and akathisia. The Curaçao Extrapyramidal Syndromes Study I. Schizophr Res. 1996; 19(2): 195–203.

4. Shin H, Chung J. Drug-Induced Parkinsonism. J Clin Neurol. 2012; 8: 15–21.

5. Bakker PR, de Groot IW, van Os J, van Harten PN. Long-Stay Psychiatric Patients: A Prospective Study Revealing Persistent Antipsychotic-Induced Movement Disorder. PLoS One. 2011; 6(10): 1–6.

